# Totally Implantable Venous Access Devices: A Randomized Controlled Trial on the Effect of Psychological Support on Quality of Life and Body Image (BI-PORT)

**DOI:** 10.3389/fpsyg.2021.703497

**Published:** 2021-11-12

**Authors:** Eleonora Pinto, Elisa Granziera, Matteo Cagol, Sandra Cappellato, Rita Alfieri, Valentina Mari, Muzio Meroni, Vittorina Zagonel, Pierfranco Conte, Pierluigi Pilati, Carlo Castoro, Francesco Cavallin, Marco Scarpa

**Affiliations:** ^1^Esophageal and Digestive Tract Surgical Unit, Veneto Institute of Oncology (IOV-IRCCS), Padua, Italy; ^2^Anesthesiology Unit, Veneto Institute of Oncology (IOV-IRCCS), Padua, Italy; ^3^Breast Surgery Unit, Veneto Institute of Oncology (IOV-IRCCS), Padua, Italy; ^4^General Surgery Unit 3, Azienda Ospedale University of Padua (AOUP), Padua, Italy; ^5^Medical Oncology Unit 1, Veneto Institute of Oncology (IOV-IRCCS), Padua, Italy; ^6^Medical Oncology Unit 2, Veneto Institute of Oncology (IOV-IRCCS), Padua, Italy; ^7^Department of Upper GI Surgery, Humanitas Research Hospital, Humanitas University, Rozzano, Italy; ^8^Independent Statistician, Solagna, Italy

**Keywords:** totally implantable venous access devices, quality of life, psychological support, body image, chemotherapy

## Abstract

**Background:** The presence of totally implantable venous access devices (TIVADs), as any permanent or semipermanent medical devices, has an impact on the quality of life (QoL) of patients. Therefore, the purpose of this trial was to evaluate the efficacy of psychological support for patients undergoing this procedure.

**Methods:** This randomized controlled trial (RCT) aimed to compare the efficacy of a psychological intervention vs. standard care on QoL in patients receiving TIVAD for chemotherapy treatment (ClinicalTrials.gov NCT02075580). The trial was conducted at the Veneto Institute of Oncology IOV-IRCCS (Padua, Italy) between October 2013 and September 2018. Participants were neoplastic adults receiving TIVAD for chemotherapy treatment for any cancer, not undergoing visible demolitive interventions, without psychopathological diagnosis and language understanding. The exclusion criteria were patients without a diagnosis of cancer, with psychopathological diagnosis, or with language misunderstanding.

**Results:** The variation of C30-QL2 and BR32-BI was not statistically different between intervention and control arms in men and women. However, the variation of C30-SF was statistically better in the intervention than control arm in men [mean difference (MD) 22.3, 95% CI 3.5 to 41.0] but not in women (MD −2.7, 95% CI −24.0 to 18.7). The variations of the other secondary outcome measures were not statistically different between intervention and control arms.

**Conclusion:** Psychological support did not show any clear advantages on global QoL and body image perception in patients at 15 days after TIVAD insertion for chemotherapy. In contrast, male patients might benefit from even a very short psychological counseling before or during chemotherapy even if they do not seem to ask for it.

## Introduction

Nowadays, totally implantable venous access devices (TIVADs) are broadly used in oncological patients when long-term venous access is needed for chemotherapy or target therapy administration (Zaghal et al., 2012; Granziera et al., 2014). There are still some debates about the TIVAD access sites (i.e., internal, external jugular, and subclavian vein) and about their insertion technique (i.e., open, percutaneous, or with ultrasound guidance). The cephalic vein approach has the advantage of the low incidence of early complications, but it has a high rate of failure. The subclavian vein is an easily accessible area, but catheters in this site have the highest risk of pneumothorax at insertion and a relatively high rate of thrombosis, vein stenosis, and catheter fatigue (Trerotola et al., 2000; Conessa et al., 2002; Granziera et al., 2014). The internal jugular approach is the most commonly used approach for tunneled infusion catheter placement with the lowest rate of venous thrombosis (Araújo et al., 2008). Usually, percutaneous access through the Seldinger technique is preferred (Biffi et al., 2001; Granziera et al., 2014; Xiong et al., 2021; Yoon et al., 2021), but in some cases, the surgical isolation of the cephalic or subclavian vein is still used. Although it is a theoretically simple surgical procedure, it can be precociously complicated by hemothorax, pneumothorax, air embolism, cardiac arrhythmia, involuntary arterial puncture, pericardial tamponade, and brachial plexus injury (Ruesch et al., 2002; Sidika et al., 2002; Granziera et al., 2014) or, lately, by thrombosis, bloodstream bacteremia, catheter malfunction, “pinch-off” syndrome, rupture, migration or embolization, superior vein cava ulceration and perforation, extravasation, pocket sepsis, and port inversion (Galloway and Bodenham, 2004; Jordan et al., 2008; Schulmeister, 2010; Granziera et al., 2014).

The presence of a TIVAD, as any permanent or semipermanent medical device, has an impact on the quality of life (QoL) of patients (Burbridge and Goyal, 2016). The WHO defines the QoL as the “individuals’ perception of their position in life in the context of the culture and value systems in which they live and in relation to their goals, expectations, standards, and concerns” (WHOQOL Group, 1995). As one of the main outcome measures in cancer treatments (Sitlinger and Zafar, 2018), QoL is a key construct involved in the evaluation of individuals after surgical treatment for TIVAD insertion. Reflecting the modification of the life attributes and conditions of patients, this multifactorial construct can be analyzed in-depth through the specific scales it is composed of. Since TIVAD is a device related to cancer, emotional functioning becomes a parameter of the conditions of patients after oncological surgery intervention. In addition, due to emotional conditions and the potential consequences on interpersonal relationships (Sitlinger and Zafar, 2018), role and social functioning should be considered subject to change after TIVAD implantation. Finally, the surgical intervention that is finalized to TIVAD insertion fits into the hospital context: it is a part of cancer treatments, and it belongs to the environment influencing the QoL of patients. Thus, the satisfaction of care is a notable part of health-related QoL involved in the environment given by this surgical intervention related to oncological cures.

In the current literature, most of the patients felt that their port had a positive impact on the management of their therapy, and they would have another port inserted if needed for future treatment (Burbridge and Goyal, 2016). In fact, ports were perceived as providing psychological benefits that included an extended sense of freedom and less invasiveness in the context of daily life and interpersonal relationships (Ryan et al., 2019). However, the port site has its importance on the subsequent impact on the lives of patients. TIVADs on the right side were observed to have a positive influence on satisfaction and acceptance of the device (Marcy et al., 2017). Moreover, arm port had less impact on daily activities and QoL (Burbridge and Goyal, 2016). In contrast, the chest ports were associated with negative features when compared to arm ports (Burbridge et al., 2016). In a recent study, a significant part of the patients reported that chest TIVAD usually interferes with their daily activities (Minichsdorfer et al., 2016). In a large multicenter study, including 720 patients in 11 French institutions, coping with a TIVAD was more difficult for younger patients than older patients, especially in terms of daily activities, body image, and private life (Marcy et al., 2017).

Body image concerns how the body is thought and felt. In addition to body appearance and shape, body size and weight evaluation, the connected reflections, intentions, and perceptions, and the related emotions and feelings are parts composing body image. Therefore, the body has a meaning consisting of all these aspects and when a surgical procedure causes different levels of changes in body appearance, different levels of body image concerns and a decline in a peculiar area of function can occur (Rhoten, 2016). A change in just one of these aspects can influence the others and consequently the whole-body image (Tylka and Wood-Barcalow, 2015). TIVAD has a diameter of about 3 cm and is placed approximately 4–5 cm below the clavicle. Externally, a small, detectable area is visible; hence, TIVAD is immediately and easily perceptible and occupies a central position in body shape. Furthermore, TIVAD implantation may involve a modification of the actions of patients and a consequent change in daily activities and body pattern (e.g., avoiding efforts with the upper body where TIVAD was implanted) or in sleeping position or even habits (e.g., dressing). Consequently, these aspects can influence the competence of patients in fulfilling their roles and may impact their interactions with social relationships and work environments (Triberti et al., 2019).

We hypothesized that TIVAD in neoplastic patients may have an impact on body image and self-representation and, thus, on QoL. Therefore, the purpose of this trial was to assess the efficacy of psychological support for patients undergoing this surgical practice.

## Materials and Methods

### Study Design

This randomized controlled trial (RCT) aimed to compare the efficacy of a psychological intervention vs. standard care on QoL in patients receiving TIVAD for chemotherapy treatment (ClinicalTrials.gov NCT02075580). The trial was conducted at the Veneto Institute of Oncology IOV-IRCCS (Padua, Italy) between October 2013 and September 2018. The trial was approved by the Ethics Committee of the Veneto Institute of Oncology IOV-IRCCS (No. 2013/12) and conducted in accordance with the Declaration of Helsinki. All participants provided written informed consent before enrollment in the study. The reporting of the study followed the Consolidated Standards of Reporting Trials (CONSORT) guidelines (Supplementary Material) (Schulz et al., 2010).

### Participants

Participants were neoplastic adults receiving TIVAD for chemotherapy treatment for any cancer, not undergoing visible demolitive interventions, without psychopathological diagnosis and language understanding. The exclusion criteria were patients without a diagnosis of cancer, with psychopathological diagnosis, or with language misunderstanding.

### Totally Implantable Venous Access Devices Implantation Technique

The TIVADs were inserted in the operating room, using rigorous sterile-barrier precautions, under local anesthesia, administering a mixture of lidocaine 1% and ropivacaine 0.5%, and employing fluoroscopic control (Granziera et al., 2014). Premedication with midazolam 0.01–0.035 mg/kg (1–3 mg) was provided in case of anxiety. A single dose of cefazoline 2 g was administered intravenously before the procedure while in beta-lactam allergic patients, vancomycin 15 mg/kg was used (Granziera et al., 2014). Then, TIVADs were flushed with 20 ml saline and filled with 5 ml of a solution containing 50 U/ml heparin to prevent clot formation and catheter blockage (Granziera et al., 2014).

Two-dimensional (2D) ultrasound imaging with a 7–12 MHz linear-array probe, which was connected to a real-time ultrasound unit (General Electric Logiq^®^ P5, GE Healthcare Clinical Systems SrL) and focused at 2–4 cm depth, was used to locate and to measure the depth and caliber of the inferior jugular vein or subclavian vein, as well as to evaluate their patency and compressibility. The catheterization of the best vessel was obtained under the continuous dynamic observation of real-time 2D images, and an 18-gauge, 7-cm needle was advanced through the skin into the vein (Granziera et al., 2014). A guidewire was then placed into the vein, the needle was removed, and the catheter was inserted. Fluoroscopy was used to confirm the correct position of the catheter at the cavoatrial junction (Granziera et al., 2014). We considered an optimal position when the catheter tip was within a 2-cm range from the lower border of the right main bronchus. Then, two skin incisions were performed: a first small one at the wire exit site from the skin, at the basis of the neck, and a second larger one, in the subclavian region, for the allocation of the TIVAD (Granziera et al., 2014). A subcutaneous tunnel over the clavicula was made to connect the catheter to the TIVAD (Granziera et al., 2014).

### Randomization

We randomly assigned participants to intervention or control arms in a 1:1 ratio, and allocation was stratified for sex. We performed the randomization using a computer-generated random assignment list. Arm assignments were sequentially numbered and placed in sealed, opaque envelopes.

### Arms

**FIGURE 1:**
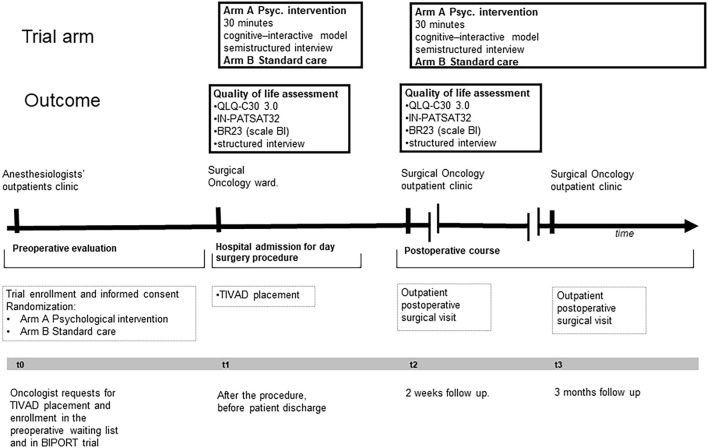
Study design.

Patients in Arm A underwent psychological support and standard care pre- and post-TIVAD implantation while those in Arm B underwent standard care. Psychological support (i.e., interactive/cognitive strategies) included as follows: cognitive/behavioral intervention (for the arm of intervention); metacognitive techniques (for the arm of intervention); and psychoeducational intervention (for the arm of intervention). In both arms, the outcome was measured with the European Organization for Research and Treatment of Cancer (EORTC) questionnaires and a written structured interview during hospital admission and 15 days after surgical intervention. The plan of the interventions is summarized in Figure 1.

### Interventions

A psychologist specialized in psychotherapy (EP) and with 5 years of experience in counseling oncological patients performed the psychological intervention. The plan of the intervention included two sessions on the day of the procedure: 30 min before the procedure (after the QoL assessment) and 30 min in the surgical ward before discharge. A third session was performed 15 days after TIVAD implantation, and further sessions could be performed from postop 15-day follow-up visit to postop 90-day follow-up visit on the request of patients.

The psychologist analyzed the self-reported evaluations of patients about body image and QoL from the preadmission questionnaire to conduct an appropriate psychological counseling session. The psychological intervention was conducted according to a cognitive–interactive model (Aricò et al., 2016; Vanaken et al., 2021). On the day of the procedure (t1), the psychologist observed the psychological processes of patients and identified worries and body distortion issues concerning their image and their self-representation. The final goal was to modify the modalities that patients might be used to prolong the self-representation as modified by TIVAD. The psychological assessment was conducted through a semi-structured interview in the first session (Harris et al., 2015; Gamsakhurdia, 2019). This approach obtained the identification of the most efficient strategies based on the body image and QoL representation and clinical course (suggestion and reorganization strategies, visualization, and other imaginative techniques, according to the intervention model) of patients. Patients were instructed on focusing on relaxing stimuli or using imaginative tactics to reduce painful stimuli due to TIVAD and on other strategies. Two weeks after TIVAD implantation (t2), in the clinic of the surgical oncology outpatients, the psychologist reminded the patients of these strategies and reinforced their efforts. Where applicable, additional interventions were performed to enhance the abilities of patients to manage the TIVAD in their daily life (active agency) (Friston, 2012). Notably, 3 months after TIVAD insertion (t3), the psychologist reminded the patients of all previous work, and together they identified both successes and failures, having a final psychological discussion on these.

### Questionnaires

The EORTC QLQ-C30 is a 30-item questionnaire and aimed to analyze the generic QoL of oncological patients (Aaronson et al., 1993; Scott et al., 2008). The EORTC IN-PATSAT32 is a 32-item questionnaire including measures of the technical skills, interpersonal skills, information provision, and availability of doctors and nurses and also satisfaction with other hospital staff, interpersonal skills and information provision, exchange of information within the care team, waiting time, hospital access, hospital comfort, and overall satisfaction with care (Brédart et al., 2005). The QLQ-BR23 module includes 23 items covering symptoms and side effects related to different treatment modalities, body image, sexuality, and future perspective of patients with breast cancer (Sprangers et al., 1996). We used, as suggested by EORTC manuals (Scott et al., 2008), the body image item to investigate the impact of psychological support on self-perception. The validation and reliability of EORTC QLQ-C30 and BR23 have been assessed by several studies in patients with different cancer types (Sprangers et al., 1996; Apolone et al., 1998; Spatuzzi et al., 2016; Sommer et al., 2020); hence, these modules were considered appropriate for this study as our sample included patients with cancer with different diagnoses and different cancer progressions.

### Outcome Measures

The primary outcome measures were general QoL (C30-QL2) and body image (BR23-BI), whereas the secondary outcome measures were emotional function (C30-EF), role function (C30-RF), social function (C30-SF), and satisfaction of care (INPATSAT32-General satisfaction). The QoL scales were evaluated as the variation from baseline (i.e., hospital admission) to postoperative (i.e., 15 days after surgery).

### Masking

Participants and researchers could not be masked to treatment allocation due to the peculiar characteristics of the intervention. In contrast, the statistician who performed the data analysis was completely blinded to treatment allocation.

### Sample Size

Any appropriate data in the literature concerning the specific topic and the type of interventions of our study were not available in the literature; thus, when designing the study, we could not base the calculation on the previously published data. The sample size was calculated to detect a standardized effect size ranging between 0.85 and 1. The calculations were performed using R 4.0 (R Foundation for Statistical Computing, Vienna, Austria) (R Core Team, 2020). Approximately 44–58 participants (22–29 in each arm) were required to have a 90% chance of detecting, as significant at the 5% level, a standardized effect size ranging from 0.85 to 1 in a parallel design. The sample size was doubled because the comparisons were stratified by sex (44–58 males and 44–58 females) and increased by 10% to consider any dropouts, leading to a final sample size of 128 participants.

### Statistical Analysis

The continuous data (scores) were expressed as mean and SD, and the categorical data were expressed as number and percentage (%). The variation from baseline (hospital admission) to postoperative (15 days after surgery) was calculated for each scale and compared between intervention and control arms using a two-sample Student’s *t*-test. Effect sizes were expressed as mean differences with 95% CIs. All analyses were stratified by sex. The reliability of the questionnaires in the study sample was assessed using Cronbach’s alpha. All tests were two-sided, and a *p*-value < 0.05 was considered statistically significant. The statistical analysis was performed using R 4.0 (R Foundation for Statistical Computing, Vienna, Austria) (R Core Team, 2020).

## Results

### Early Termination

**FIGURE 2:**
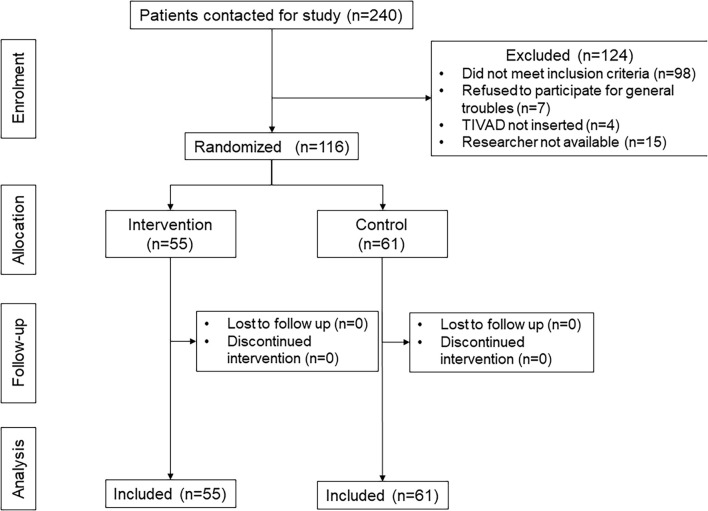
Flowchart of patient inclusion.

In June 2017, the Surgical Unit was dismantled, and this led to the decision to terminate the study earlier. The Ethics Committee of the Veneto Institute of Oncology IOV-IRCCS approved the early termination in January 2018. Among 240 contacted patients between October 2013 and September 2018, 98 did not meet the inclusion criteria, 7 refused to participate for general troubles, and in 4 patients, TIVAD was not inserted, and for 15 patients, any researcher was available. Finally, 116 participants (median age 61 years; 50 males and 66 females) were enrolled in this study. No contamination between arms occurred, and all participants received the allocated intervention (Figure 2). Participant characteristics are shown in Table 1. Cronbach’s alpha indicated good reliability of the questionnaires in our sample (C30 α = 0.86, BR23 α = 0.85, INPATSAT32 α = 0.98).

**TABLE 1:** Patient characteristics.

	**Males (*n* = 50)**		**Females (*n* = 66)**	
	**Intervention arm (*n* = 25)**	**Control arm (*n* = 25)**	**Intervention arm (*n* = 30)**	**Control arm (*n* = 36)**
Age, years: mean (SD)	63 (9)	63 (13)	59 (12)	58 (12)
**Tumor stage:**				
I-III	7 (28%)	3 (12%)	4 (13%)	8 (22%)
IV	18 (72%)	22 (88%)	26 (87%)	28 (78%)
**Chemotherapy at TIVAD placing:**				
• Before starting	10 (42%)	13 (52%)	10 (33%)	14 (39%)
• First line	3 (12%)	6 (24%)	7 (23%)	10 (28%)
• Second line	11 (46%) (miss = 1)	6 (24%)	13 (44%)	12 (33%)
**Intention to treat:**				
• Neoadjuvant	0 (0%)	2 (8%)	1 (3%)	8 (23%)
• Adjuvant	4 (17%)	5 (20%)	12 (40%)	4 (11%)
• Palliative	19 (83%) (miss = 2)	18 (72%)	17 (57%)	23 (66%) (miss = 1)
Previous surgical treatment	14 (56%)	17 (68%)	22 (73%)	21 (58%)
Previous PORT removal	0	0	1 (3%)	2 (6%)
Complications after PORT placement	0	0	0	1 (3%)

### Primary Outcome Measures

**FIGURE 3:**
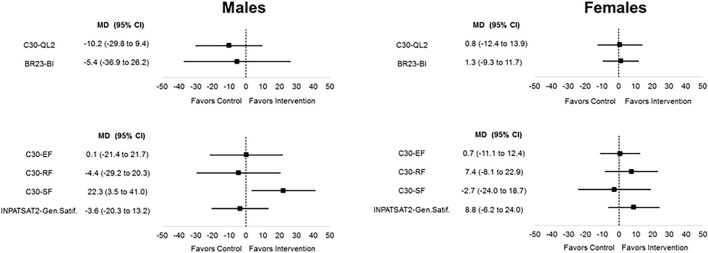
Primary and secondary outcome measures stratified by sex: variation from baseline (hospital admission) to postoperative (15 days after surgery).

As shown in Figure 3, the variation of C30-QL2 was not statistically different between intervention and control arms in males [mean difference (MD) −10.2, 95% CI −29.8 to 9.4] and in females (MD 0.8, 95% CI −12.4 to 13.9) (Figure 3). The variation of BR32-BI was not statistically different between intervention and control arms in males (MD −5.4, 95% CI −36.9 to 26.2) and in females (MD 1.3, 95% CI −9.3 to 11.7) (Figure 3). The numerical results are reported in Appendix Table 1.

### Secondary Outcome Measures

As shown in Figure 3, the variation of C30-SF was statistically better in the intervention than the control arm in males (MD 22.3, 95% CI 3.5 to 41.0) but not in females (MD −2.7, 95% CI −24.0 to 18.7). The variations of the other secondary outcome measures were not statistically different between intervention and control arms (Figure 3). The numerical results are reported in Appendix Table 1.

## Discussion

Currently, TIVADs are broadly used in oncological patients when long-term venous access is requested for the administration of chemotherapy or intravenous targeted agents (Zaghal et al., 2012). However, the presence of a TIVAD interferes with the activities of daily living of patients (Tylka and Wood-Barcalow, 2015), thus, has an impact on the QoL of patients (Burbridge et al., 2016), and can have an impact on body image due to the presence of the subcutaneous swelling in the subclavian region. The aesthetical appearance might affect more female patients as suggested by a recent study that reported significantly worse scores related to patient satisfaction in women (Marcy et al., 2017). Therefore, we hypothesized that TIVAD in neoplastic patients may have an impact on body image and self-representation and, thus, on QoL. Therefore, the purpose of this trial was to evaluate the efficacy of psychological support for patients undergoing this surgical practice.

Our findings showed that the variation of C30-QL2 was not statistically different between intervention and control arms neither in males nor in females. In our previous trial, psychological counseling managed to reduce the impairment in QoL and quality of sleep in patients undergoing esophagectomy for cancer (Scarpa et al., 2017). Similarly, a Canadian study on head and neck cancer observed that the short-term psychoeducational coping strategies intervention ameliorated physical and social functioning and global QoL, while they lessened fatigue, sleep disturbance, and depressive symptoms (Vilela et al., 2006). The lack of effect on global QoL in this study might be either due to the truly short duration of the intervention or due to the strict focus on TIVAD acceptance. In fact, these patients might be more focused on and worried about the whole oncological situation than on the mere TIVAD insertion and its effect.

Rather unexpectedly, the psychological support did not obtain any statistically significant variation in terms of the score of body image neither in males nor in females.

This literature is particularly focused on body image in cancers affecting organs directly implied in sexual life or reproductive system, such as breast cancer or prostate cancer, whose treatment effects have substantial psychological and social connotations (Bowie et al., 2020; Morales-Sánchez et al., 2021). In a very recent study on a web-based psychological intervention aimed to alleviate body image-related distress after breast cancer treatment through a single-session, self-compassion-focused writing activity, significantly greater body appreciation and self-compassion were observed in the interventional arm (Sherman et al., 2018).

Different models of the psychological interventions on patients and survivors of breast cancer (e.g., psychoeducational program, psychosocial intervention, psychotherapy, physical-activity based approach, art therapy, dance/movement activities, mindfulness-based stress reduction treatment, and metacognition group therapy) have been shown to be effective with medium effect size (Sebri et al., 2021) or effect sizes ranged from small to large (Lewis-Smith et al., 2018). These studies pointed out that approaches stressing the peculiar experiences of individuals and their narrations, as well as in psychotherapy, are supposed to be associated with larger effects on body image improvement (Lewis-Smith et al., 2018; Sebri et al., 2021).

To our knowledge, the literature does not offer any other studies on psychological interventions which may be effective on body image in patients with cancer receiving TIVAD; thus, the interpretation of our findings only allows some speculations. First, we underlined that the psychological counseling aimed to relieve the burden of the presence of a subcutaneous swelling in the subclavian region due to port insertion. Hence, such psychological support helped patients in coping with this aspect but did not change the fact that the swelling was there and still visible. Moreover, the body changes are also due to chemotherapies, and the global modification in self-image could also be perceived after some months. Furthermore, the outcome measure was assessed very shortly after surgery when not only the swelling but also the dressing, the fresh scar, and the postoperative pain were a clear reminder of the port presence.

Of note, our intervention was associated with improved social functioning in male participants, while this was not evident in females. Since TIVAD is placed next to the breast, sexual functioning is among the activities and functions that can be altered, with consequent emotions and feelings that a brief psychological intervention can only partially manage (Satinsky et al., 2012). The proximity of TIVAD to the breast and the possible changes in actions regarding this female sex organ could increase interoceptions and influence the sensation of an internal pathological state. The impact on such important aspects can harm the perception of being able to have a role in social environments. Moreover, in a recent study on the effect of a 1-week educational program on caregivers of patients with cancer, female patients more frequently reported a need for psychological counseling, group conversations, nutritional counseling, and recreational stay (Gjerset et al., 2019). Therefore, we speculated that female patients might already have faced up to cancer and chemotherapy impact due to their capacity of asking for support. In contrast, unsolicited psychological counseling, coming from the trial, might have given male patients the unexpected support that might have helped them to improve their social functioning. In any case, these results suggest that male patients might benefit from even a very short psychological counseling before or during chemotherapy.

According to our findings, TIVAD is likely to be a feature leading to the typification of patients with cancer (Kelly, 2015) as the device points out that there is a difference in that body, with effects on the interoceptions, intentions, and emotions. Hence, even when supported with professional psychological care, patients with TIVAD can experience a low role and emotional functioning, since their activities and functioning imply the perception of the presence of this device. The main limitation of this study is the early termination and its consequences. In June 2017, the Surgical Unit was dismantled, and this event suggested us to terminate the study earlier. The early termination led to a sample size lower than planned. Nonetheless, the initial sample size calculation suggested that the achieved sample size was sufficient to detect a potential difference of 10% of the QoL score, which is the minimal difference that could be qualified as clinically meaningful and significant (Scott et al., 2008). Hence, the actual findings imply that our intervention might not provide clinically significant benefits on the target outcomes. Open questions remain about the impact of different interventions (i.e., different aims or content of the psychological support), the opportunity of different implementations (i.e., prolonged duration or inclusion of group support), and the appropriateness of other outcome measures [i.e., social functioning or daily activities (Burbridge et al., 2016; Minichsdorfer et al., 2016)].

According to our findings, the psychological counseling for patients undergoing TIVAD implantation may focus on the aspects supposed to be impaired by the device, e.g., self-esteem, and may prevent other possible consequences, such as anxiety related to chemotherapy as reminded by TIVAD and fear of recurrence. In fact, this literature suggests that self-esteem, as the feeling of satisfaction with oneself based on the evaluation of characteristics of an individual, can be a protective factor for anxiety due to the negative opinions of others. In fact, self-esteem is related to body image by the association between body mass index, body shape, and negative evaluation (Bowie et al., 2020; Morales-Sánchez et al., 2021). The connection between body image, self-esteem, and fear of negative evaluation suggests that such factors may affect the QoL of patients and thus remains a potential target of an intervention (Ahadzadeh et al., 2018).

Moreover, other aspects such as sexual functioning or anxiety related to an inserted device may be investigated and improved as possible specific factors in the body image of patients with cancer with TIVAD. This literature underlines the relevance of sexual functioning as part of body image and body image distortions and suggests the opportunity for proper preventive intervention. When failing to assess sexual functioning as one of the main aspects of body image, the psychological intervention may not affect sexual functioning strictly related to body image and may not be effective (Sebri et al., 2021).

According to this, other outcome measures such as anxiety, sexual functioning, and self-esteem could be considered with appropriate tools. Patients could also benefit from group psychological interventions since peers can reflect on different experiences, ways of considering the physical changes, feelings, and emotions about TIVAD, thus helping to maintain a proper body image in patients with cancer. In group interventions, the stage of treatment of participants could be also considered to construct a targeted intervention (Lewis-Smith et al., 2018).

According to the abovementioned studies, some other features can be considered for the psychological intervention concerning TIVAD implantation. First, the intervention should be based on an approach highlighting the personal experiences of patients. This approach considers a 5–14-week-long psychological intervention, which could attain long-lasting improvements (Lewis-Smith et al., 2018). Therefore, the limited effectiveness of our intervention suggests that the support should cover a period longer than 15 days after TIVAD implantation and further sessions have to be implemented even after 3 months, e.g., extending the psychological intervention to the next tumor restaging or the first oncological follow-up. In any case, it should not be suspended until one treatment phase involving TIVAD is concluded. Moreover, the psychological programs should be structured not only focusing on biomedical and disease aspects but also targeting broader contents, reducing so-called false beliefs and including relaxation training, besides enhancing problem-solving and adaptive skills conducted in our intervention (Lewis-Smith et al., 2018). Furthermore, the evaluation of body image at cancer diagnosis may give the baseline for analyzing changes in body image during cancer treatments.

## Conclusion

The psychological support did not show any clear advantages on global QoL and body image perception in patients at 15 days after TIVAD insertion for chemotherapy. In contrast, male patients might benefit from even a very short psychological counseling before or during chemotherapy even if they do not seem to ask for it. Finally, TIVAD insertion is a quick and easy procedure for the surgeon, but it is a heavy burden for patients who would need comprehensive psychological treatment.

## Data Availability Statement

The raw data supporting the conclusions of this article will be made available by the authors, without undue reservation.

## Ethics Statement

The studies involving human participants were reviewed and approved by Ethics Committee of the Veneto Institute of Oncology IOV-IRCCS (number 2013/12). The patients/participants provided their written informed consent to participate in this study.

## Author Contributions

EP, FC, and MS gave substantial contributions to conception and design, acquisition of data, and analysis and interpretation of data, and involved in drafting the manuscript. EG and SC gave substantial contributions to the acquisition of data and interpretation of data, and involved in critical revising for important intellectual content. FC gave substantial contributions to the analysis and interpretation of data, and involved in drafting the manuscript. MC, SC, RA, and VM gave substantial contributions to the acquisition of data and involved in critical revising for important intellectual content. MM, VZ, PC, PP, and CC gave substantial contributions to the conception and design, and interpretation of data, and involved in critical revising for important intellectual content. All authors read and approved the final manuscript.

## Conflict of Interest

The authors declare that the research was conducted in the absence of any commercial or financial relationships that could be construed as a potential conflict of interest.

## Publisher’s Note

All claims expressed in this article are solely those of the authors and do not necessarily represent those of their affiliated organizations, or those of the publisher, the editors and the reviewers. Any product that may be evaluated in this article, or claim that may be made by its manufacturer, is not guaranteed or endorsed by the publisher.

## Appendix

**APPENDIX TABLE 1:** Primary and secondary outcome measures stratified by sex: variation from baseline (hospital admission) to postoperative (15 days after surgery).

**Outcome measure**	**Scale**	**Variation from baseline (hospital admission) to postoperative (15 days after surgery)**							
		**Males**				**Females**			
		**Intervention arm: mean (SD)**	**Control arm: mean (SD)**	**Intervention vs. control arm: MD (95% CI)**	***p*-value**	**Intervention arm: mean (SD)**	**Control arm: mean (SD)**	**Intervention vs. control arm: MD (95% CI)**	***p*-value**
Primary	C30-QL2	−2.8 (8.7)	7.4 (25.1)	−10.2 (−29.8 to 9.4)	0.27	−6.9 (22.0)	−7.7 (13.8)	0.8 (−12.4 to 13.9)	0.91
	BR23−BI	−1.2 (9.7)	4.2 (30.2)	−5.4 (−36.9 to 26.2)	0.69	−1.0 (14.2)	−2.3 (14.8)	1.3 (−9.3 to 11.7)	0.81
Secondary	C30−EF	3.8 (21.3)	3.7 (25.4)	0.1 (−21.4 to 21.7)	0.99	−5.1 (16.2)	−5.8 (15.4)	0.7 (−11.1 to 12.4)	0.91
	C30−RF	−4.4 (16.0)	0.0 (31.2)	−4.4 (−29.2 to 20.3)	0.7	−0.9 (20.2)	−8.3 (23.6)	7.4 (−8.1 to 22.9)	0.34
	C30−SF	5.6 (22.4)	−16.7 (20.4)	22.3 (3.5 to 41.0)	0.02	−7.4 (39.7)	−4.7 (16.6)	−2.7 (−24.0 to 18.7)	0.8
	INPATSAT32-General satisfaction	0.0 (11.8)	3.6 (17.3)	−3.6 (−20.3 to 13.2)	0.64	3.1 (17.9)	−5.7 (20.8)	8.8 (−6.2 to 24.0)	0.24

*MD, mean difference.*
